# Environmental Impact Assessment, Human Health and the Sustainable Development Goals

**DOI:** 10.3389/ijph.2022.1604420

**Published:** 2022-01-31

**Authors:** G. Gulis, N. Krishnankutty, E. R. Boess, I. Lyhne, L. Kørnøv

**Affiliations:** ^1^ Unit for Health Promotion Research, University of Southern Denmark, Esbjerg, Denmark; ^2^ COWI, Lyngby, Denmark; ^3^ Danish Centre for Environmental Assessment, Aalborg University, Aalborg, Denmark

**Keywords:** population health, SDGs, impact assessment, EIA, indicators

## Abstract

**Objectives:** Developmental processes influence the determinants of health and, consequently, human health. Yet, assessing human health impacts in impact assessment, with exception of health impact assessment, is still rather vague. Inclusion of Sustainable Development Goal indicators in environmental impact assessment (EIA) is an opportunity to enhance addressing human health in EIA practices.

**Methods:** We reviewed a list of health-related targets and indicators for SDGs as defined by the Institute of Health Metrics and Evaluation (IHME) in Seattle, WA, United States with the aim of identifying those to be suggested as outcome indicators within EIA.

**Results:** Among 42 health-related indicators, we identified 17 indicators which could be relevant for impact assessment procedures and categorized them into three groups: 1) direct health indicators (e.g., under five mortality). 2) complex indicators (e.g., cancer). 3) environmental determinant indicators (e.g., mean PM_2.5_).

**Conclusion:** All 17 indicators can be employed to improve quantification assessing human health impacts and bring SDGs into EIA processes. Though our assessment has been conducted for Denmark and the set of suggested indicators could be different for contexts in other countries, the process of their identification can be generalized.

## Introduction

Environmental impact assessment (EIA), typically understood as project level assessment of a broad set of environmental impacts, has a long-term success history since its first statutory introduction in 1969 [1]. Since then, most countries introduced EIA into their legislation and made it a central tool to improve developmental processes and inform of their impact on environment [2]. Seeing as EIA is a widespread mandatory form of impact assessment, it represents an important arena for uncovering potential human health impacts as part of a broader concept of environment. In this way, the EIA complements Health Impact Assessment (HIA), which is usually a voluntary assessment focusing on potential health risks and benefits. Despite the indivisible link between environment and health, impacts on human health have not been a focus of EIA in the early decades and even health determinants (with the exception of environmental determinants) were rare and narrowly discussed within EIA reports [3]. A major legislative change to this practice occurred in 2014, when the European Commission, via an amendment to the directive (Directive 2014/52/EU), formally introduced impact on human health as a mandatory impact to assess within EIA [4]. Denmark, as other European Union countries, implemented the Directive to national legislation making assessment of population health impacts mandatory within EIA [5]. This act provided a requirement to address health more in depth in assessment processes and opened a space for research on tools for assessment and quantification of health impacts. Yet, the historical scope of EIA coupled by a lack of involvement of health expertise in conducting assessments led to the recognition of rather limited inclusion of health in EIA [6–8]. In 2018–19, a reference document was formulated to better address human health in EIA, by the joint venture of International Association for Impact Assessment (IAIA) and the European Public Health Association (EUPHA) [9]. This document constituted that the identification of relevant health impacts, the development of proper indicators to measure them and the assessment of their impacts is a rather complex task. Despite of the guidance provided, the methodological complexities (both qualitative and quantitative methods) are challenging for the non-health professionals who conduct the screening, scoping and especially risk appraisal procedures in EIA [10]. A Danish innovation project “Digitally Supported Environmental Assessment for Sustainable Development Goals—DREAMS” addresses among other issues, inclusion of human health into EIA and Strategic Environmental Assessment (SEA) linking the whole process to the United Nations Sustainable Development Goals (SDGs) [11]. The project aims to explore whether the SDG indicators can be used as target indicators within EIA and SEA. In this article, we focus solely on the project-level assessment within EIA.

The SDGs have provided a global development framework for sustainable development universally applicable to all countries. The 17 goals, 169 targets and 247 unique indicators can be perceived not only as an ambitious set of measures to guide development, but also as an opportunity to compare countries and harmonize processes across different contexts. One of the primary issues with their implementation is how best to operationalize the SDGs in national and local developmental processes, hereunder including environmental assessment procedures. Some authors have proposed that SDGs and environmental assessment can mutually benefit each other, such that SDGs help to provide a sustainable orientation for environmental assessment and bring sustainability objectives into decision-making processes, while environmental assessments simultaneously provide a structured and universally exercised process for measuring SDG fulfillment [12–14]. Yet, the need for localizing the globally developed SDGs remains also a challenge when considering their integration into environmental assessment, and although experimentation in linking SDGs to EIA is beginning to emerge within practice, the integration is predominantly superficial and seemingly disconnected from the potentials constituted through research [12, 15]. Literature [16] and practical application cases recently published [17, 18] have likewise raised issues with the implementation of SDGs in health impact assessment. There is therefore a need for literature to assist in operationalizing the SDGs and guide practice to encourage a productive utilization within impact assessment.

In attempts to better operationalize the SDGs and understand their potential function as decision-support tools, conceptual frameworks to link SDGs with environmental assessment have been developed and published [12, 13]. Inclusion of SDG indicators as target indicators within impact assessment processes seems to be therefore mutually complementary [14]. Some studies have also suggested that the SDGs address sustainability parameters that if applied to environmental assessment, may make for more comprehensive assessments also able to remain current with sustainability agendas [19, 20]. While implying health as a parameter with the potential for improved assessment, few studies have yet specifically focused on elaborating the overlap between health determinants and SDGs, nor have they addressed SDG indicators as a way to support these assessments [17, 18].

**FIGURE 1:**
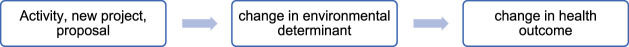
Framework of selection of health indicators. Environmental Impact Assessment, Human Health and the Sustainable Development Goals, Denmark, 2021.

The aim of our work and this manuscript is to investigate which SDG indicators can support the assessment of health impacts in EIA processes as measures of final health outcomes related to the assessed project. Our focus is on Denmark predominantly, yet we believe the process allows for the generalization to other contexts as well. Our conceptual framework for selection of indicators can be described by this simplified pathway (Figure 1):

## Methods

Concrete tools or selection criteria are currently unavailable for selecting or prioritizing relevant health-related SDGs for EIA. Our focus within this work is on project-level EIA; therefore not all SDG health-related targets and indicators may be applicable to review in all EIA projects, as some SDGs and corresponding indicators may pertain more to strategic development than is addressed through a project-level EIA. Since only the indicators substantialize the content of the SDGs and make contributions measurable, it is necessary to select relevant indicators for EIA given a criteria-based approach, which will also aid in narrowing down the 232 SDG indicators that are currently developed on the global plan.

### Criteria to Select Relevant Environmental Health SDG Indicators

Though SDGs are predominantly designed for countries and regions, predefined indicators can be used as guidelines for addressing health aspects in EIA. As a first step, all SDG indicators presented by UNSTATS were considered [21]. The goals, however, are very general and not only applicable to health aspects. In the second step, health-related targets and indicators for SDGs were narrowed down to health-related indicators within a specific country, namely Denmark, by looking at availability of data on indicators in national statistics. Using the metadata of Denmark defined by the Institute of Health Metrics and Evaluation (IHME) in Seattle, WA, United States [22] and Statistics Denmark, health targets and indicators were identified. The third step aimed to identify outcome indicators relevant for EIA. Through an internal expert consultation, we identified those indicators, which can be linked to developmental processes and therefore used within impact assessment. The internal expert consultation consisted of five experts within the field of public health, HIA, environmental health and EIA. The consultation was guided by following protocol:

• We looked at the listing of typology of investment projects subjected to EIA and discussed whether a specific type of investment project can have an impact on environmental determinants of health and selected health indicators.
• We appraised whether the indicator addresses a health outcome or an environmental determinant of health that can be used as part of a causal pathway description. Those measuring health outcomes were classified as either direct or complex health outcomes.

**FIGURE 2:**
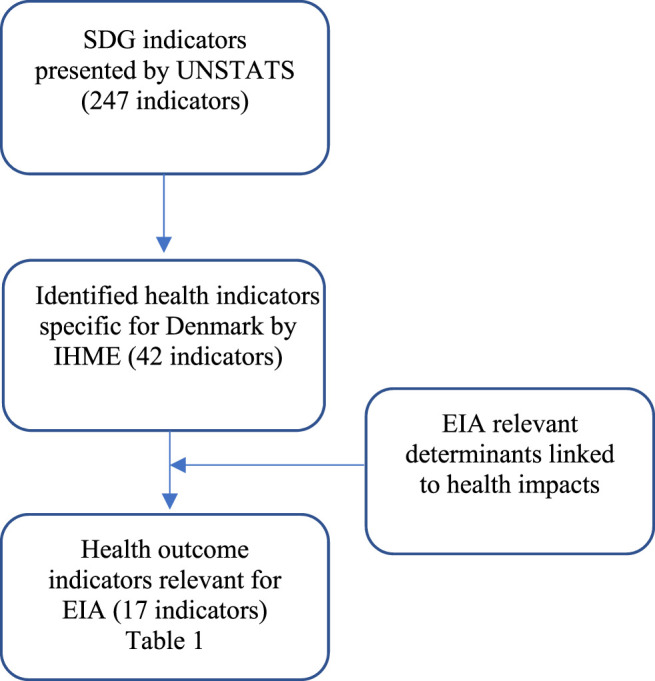
Schematic diagram to select and link SDG health indicators with EIA. Environmental Impact Assessment, Human Health and the Sustainable Development Goals, Denmark, 2021.

The direct health indicators are considered indicators that can be used directly in assessment, whereas the complex indicators may require further break down into more specific health outcomes before being used within EIA. Figure 2 describes the selection flow.

## Results

The analysis of the criteria-based approach to select and link SDG health indicators within EIA identified a wide range of indicators that relate to the health aspects, i.e., physical health; well-being; access to safe amenities; environmental impacts. From among 42 health-related indicators, 17 indicators were identified to be relevant and two indicators potentially relevant for EIA. The list of indicators considered relevant is in Table 1.

**TABLE 1:** List of identified indicators. Environmental Impact Assessment, Human Health and the Sustainable Development Goals, Denmark, 2021.

	SDG health INDICATORS	Direct	Complex	Environmental	Environmental risk factors (relevant to EIA)
1	3.2.1. Under-5 mortality	x			Water pollution (micro-pathogens causing diarrhoea), air pollution (carbon monoxide and fine particulate matter causing pneumonia), high temperature and humidity
	3.4.1. Non communicable disesas mortality		x		
2	a. Cardiovascular disease	x			Exposure to urban air pollution (fine particulate matter), metals (lead, cadmium, arsenic)
3	b. Cancer		x		Outdoor air pollution, heavy metals, water pollutants (Organic and inorganic chemicals derived from industrial, commercial and agricultural activities, and in particular from waste sites, nitrites and nitrates, radionuclides and asbestos)
4	c. Diabetes	x			Air pollution, physical activity environment and roadways proximity, polluted air, soil, water
5	d. Chronic respiratory disease	x			Indoor and outdoor air pollutants (Particulate NO_2_, S02, 03) Inorganic dusts (chalk and talc), fumes and gases (metal, chlorine, SO_2_, H_2_S, styrene, polyvinyl chloride/methyl, methacrylate)
6	3.4.2. Suicide mortality	x			
7	3.6.1. Road injury mortality		x		Roadways proximity
8	3.9.1. Air pollution mortality				Indoor and outdoor air pollutants (Particulate NO_2_, S02, 03) Inorganic dusts (chalk and talc), fumes and gases (metal, chlorine, SO_2_, H_2_S, styrene, polyvinyl chloride/methyl, methacrylate)
9	3.9.2. Water, sanitation and hygenie (WaSH) mortality		x		Water pollutants (Organic and inorganic chemicals derived from industrial, commercial and agricultural activities, and in particular from waste sites, nitrites and nitrates, radionuclides and asbestos)
10	3.9.3. Unintentional poisoning mortality		x		
11	6.1.1. Usage of unsafe water, summary exposure value (SEV)			x	
12	6.2.1a. Unsafe sanitation (SEV)			x	
13	6.2.1b. Unsafe hygiene			x	
14	7.1.2. Household air pollution			x	Indoor air pollutants (particulate matter)
15	8.8.1. Disability adjusted life years (DALY) due to occupational burden		x		
16	11.6.2. Mean PM2.5			x	Particulate matter

The identified relevant health indicators are categorized to reflect how the health indicators are in relation to project activities in EIA, and how the outcome indicators constitute a consideration of human health in EIA. Indicators are categorized as direct indicators, complex indicators and environmental indicators. The direct indicators are the indicators that are affected during either the operational or construction phase of the developmental activity. When calculating the impacts, the direct indicators directly describe the baseline values and estimate the impact. These are, in most cases, part of national demographics or health statistics. They can often be characterized by a code according to International Classification of Diseases—ICD code [23]. The complex indicators are characterized either by merging many determinants into one health outcome, covering a group of individual diseases (e.g., cancer) or by being a composite indicator (e.g., DALY). To apply complex indicators within EIA, a human health expertise is required to estimate cumulative impact derived from the selected indicator, which, in some cases, could also be considered sub-indicators. The third classification is environmental determinant indicators, which describe environmental characteristics or the target area of the assessed activity. Their application to assess human health impacts within EIA requires linkage to one of the mentioned direct or complex health indicators via casual pathways.

The two potentially relevant indicators are natural disasters and vulnerability to poverty (SDG indicator number 1.5.1) and maternal mortality (3.1.1). The first one is considered potentially relevant for EIA depending on the subject of the development activity, as well as the geographical and social conditions of the population in the target area of activity. Maternal mortality can be relevant for use in EIA, if the activity influences either social conditions, such as education, or the health system and access to health services in a target area. Environmental factors, which could be a part of the relevant risk factors for maternal mortality, are directly addressed by enlisted indicators.

## Discussion

Addressing human health within EIA processes is a window of opportunity to strengthen the human health agenda within developmental processes at all levels (global-local as continuum). EIA, contrary to health impact assessment (HIA), is a statutory process in most countries of the world and, as such, directly links both to governance and to economic decision making (e.g., financial sectors and loans). Inclusion of SDG indicators as outcome indicators in assessment processes could prove to be mutually complementary. It may better align new projects with SDGs and, at same time, offer a more standardized approach to conducting population health assessment that is more encompassing of the international and normative policies defining future development. A conceptual framework presented by Kørnøv et al. [12] divides SDG-integration into various levels, differentiating between non-integration, conservative integration and radical integration. Drawing upon SDG indicators when measuring impact on health parameters in EIA would help to substantiate SDG-integration within, at minimum, the third level through conservative integration, in which SDG indicators support scoping and defining significant impacts. However, using SDG-derived health indicators to actively test project impacts or as elements of decision-making throughout the process could allow for the navigation into higher levels of integration.

Another issue where inclusion of SDG indicators might help to address human health impacts within EIA is availability of data. Impact assessment processes are often restricted by a lack of “ready-to-use” data and require specific data collection prolonging the time of assessment [24]. SDG indicator values are collected on national levels and might be available also on a regional or local level. Countries are developing their own data collection frameworks including surrogate indicators as in Denmark for example the “Vores Mål” report [25]. Naturally, these indicators can well be used to describe the baseline levels within EIA reports before implementation of the project. Such a set of indicators can be employed in screening, scoping and risk appraisal phases of assessment processes.

Having a set standard of health outcome indicators for assessment of human health impacts within EIA (but also other types of impact assessments) could invite for a discussion on possibilities to standardize impact assessment process through standardization of indicators. Typology of projects subjected to EIA are usually described in annexes of national legislation providing broad, but to some extent, pre-defined types of developmental activities (e.g., transportation infrastructure projects). On the other hand, national health policies (programs) usually pre-define priority areas of human health measures (e.g. cardiovascular disease, cancer, diabetes). What remains to be done is linking the two ends of impact assessment; having a well-defined (standard) set of health outcome indicators could substantially enhance quality of assessment processes. Such standard set of indicators could also contribute to important workforce issues. Human health expertise should always be part of the impact assessment process and having a standard set of outcome indicators can better specify what kind of expertise is necessary to involve.

### Conclusion

Impact assessment processes became a significant and positive tool to protect the environment as well as human health, even though the original scope in legislation was oriented towards environmental protection. Recent changes in legislation especially within Europe opened a window of opportunity for improved targeting of human health impacts within EIA. At the same time, the global effort towards the achievement of SDGs as a guiding policy ambition opens the issue of integration of SDGs into impact assessment processes. Our short paper outlines possibilities and potential benefits of such integration on the indicator level and also proposes those indicators that may be relevant for consideration in Danish EIA practices.
